# Surgical options for patients with early-stage breast cancer and pathogenic germline variants: an oncologist perspectives

**DOI:** 10.3389/fonc.2023.1265197

**Published:** 2023-09-14

**Authors:** Hikmat Abdel-Razeq

**Affiliations:** ^1^ Department of Internal Medicine, King Hussein Cancer Center, Amman, Jordan; ^2^ School of Medicine, The University of Jordan, Amman, Jordan

**Keywords:** hereditary breast cancer, risk-reducing surgery, ipsilateral breast tumor recurrence, ATM, CHEK2, BRCA, PALB2, TP53

## Abstract

Breast cancer continues to be the most common cancer diagnosed among women worldwide. Family history of breast cancer is frequently encountered, and 5-15% of patients may carry inherited pathogenic germline variants, identification of which can be helpful for both; patients themselves and their unaffected close relatives. The availability and affordability of molecular diagnostics, like next generation sequencing (NGS), had resulted in wider adoption of such technologies to detect pathogenic variants of cancer-predisposing genes. International guidelines had recently broadened the indications for germline genetic testing to include much more patients, and also expanded the testing to include multi-gene panels, while some professional societies are calling for universal testing of all newly diagnosed patients with breast cancer, regardless of their age, personal or family history. The risk of experiencing a contralateral breast cancer (CBC) or ipsilateral recurrence, is well known. Such risk is highest with variants like *BRCA1* and *BRCA2*, but less well-studied with other less common variants. The optimal local therapy for women with *BRCA*-associated breast cancer remains controversial, but tends to be aggressive and may involve bilateral mastectomies, which may not have any survival advantage. Additionally, surgical management of unaffected women, known to carry a pathogenic cancer-predisposing gene, may vary from surveillance to bilateral mastectomies, too. The oncological safety, and the higher satisfaction of unaffected women and patients with new surgical techniques, like the skin-sparing (SSM) and nipple-sparing (NSM) mastectomies, eased up the process of counselling. In this review, we address the oncological safety of less aggressive surgical options for both; patients and unaffected carriers.

## 1 Introduction

Breast cancer is the most common cancer worldwide and is considered one of the leading causes of cancer-related mortality in both developed and developing countries. In 2020, about 2.3 million women were diagnosed with breast cancer worldwide and 685,000 died of their disease (1). In 2023, almost 300,000 women will be diagnosed with breast cancer in the U.S alone (2). Almost one in five patients with newly diagnosed breast cancer report a family history of breast cancer (3–5). However, smaller fraction may be attributed to an inherited cancer-predisposing gene, mostly in *BRCA1* or *BRCA2* (6). Based on one meta-analysis, the estimated mean cumulative risk for developing breast cancer by age 70 for carriers of the *BRCA1* variant is 57%, whereas the risk for carriers of the *BRCA2* variant is a little lower at 49% (7). However, other studies reported higher cumulative breast cancer risk (72%) to age 80 for *BRCA1* and 69% for *BRCA2* carriers (8). The extent to which other pathogenic variants, like *CHEK2*, *PALB2*, *ATM*, *TP53*, are associated with breast cancer susceptibility varies significantly (9, 10).

Molecular diagnostics, like next generation sequencing (NGS), is becoming affordable and is widely utilized to detect variants in cancer predisposing genes (11, 12). For patients without *BRCA1/2* variants, breast-conserving surgery (BCS), with or without neoadjuvant chemotherapy, followed by radiation therapy, is the treatment of choice for most patients; it offers similar survival to that of mastectomy (13–16). More recent study claimed even better survival outcome with BCS followed by radiation therapy, compared to mastectomy (17–20). In a recent study that used the Surveillance, Epidemiology and End Results (SEER) database which identified 205,788 women with breast cancer diagnosed from 1988 to 2018, patients who underwent BCS and radiotherapy had higher competing risk of breast cancer recurrence (adjusted hazard ratio [HR]: 1.996, 95% CI: 1.925-2.069, p<0.001) and lower competing risk of breast cancer-specific death (BSD) when compared to mastectomy (adjusted HR: 0.584, 95% CI: 0.572-0.597, p<0.001) (21). Another study that also used the SEER database reached almost similar conclusions (22). Additionally, BCS provides better quality of life; a recent study concluded that patients treated with BCS were more satisfied with their cosmetic outcome compared to those who had mastectomy with or without reconstruction (23).

In this review, we discuss surgical treatment options for patients with breast cancer known to have a high-penetrant cancer-predisposing gene, like the *BRCA1* and *BRCA2*, and address the oncological safety of less aggressive surgical options, for both patients and unaffected carriers.

## 2 The prevalence of germline mutations

Depending on population studied and method of testing, 5-15% of breast cancer patients are carriers of one of the increasingly recognized hereditary predisposition genes. Multiple studies have evaluated the prevalence of pathogenic (PV) or likely pathogenic variants (LPV) in breast cancer patients; majority of such studies were retrospective and from single institution. In a large industry sponsored study, over 35,000 women with breast cancer underwent germline genetic testing with a 25-gene panel. PV/LPVs were detected in 9.3% of women tested; 51.5% were in genes other than *BRCA1* or *BRCA2*, including *CHEK2*, *ATM* and *PALB2*. Rates were significantly higher among younger women aged < 40 years (24). In another study, all women 20 years of age or older diagnosed with breast (or ovarian cancer) in the state of California and Georgia in 2013 and 2014, and reported to the SEER registries were reviewed. Over 77,000 patients with breast cancer were included; almost 25% of them had genetic test results. Pathogenic variants were mostly in *BRCA1* (3.2%), *BRCA2* (3.1%), *CHEK2* (1.6%), *PALB2* (1.0%) and *ATM* (0.7%) (25).

We recently reported our experience on 1,310 non-Western patients diagnosed with breast cancer. Patients were tested as per the National Comprehensive Cancer Network (NCCN) guidelines. Age ≤ 45 years was the most common indication for testing, while positive family history of breast, ovarian, pancreatic or prostate cancers, and triple-negative disease were among other frequent indications. Among the whole group, 184 (14.0%) patients had PV/LPVs; only 90 (48.9%) were in *BRCA1* or *BRCA2*, while 94 (51.1%) others had pathogenic variants in other genes; mostly in *APC*, *TP53*, *CHEK2* and *PALB2*. Mutation rates were higher among patients with positive family history (*p*=0.009); especially if they were 50 years or younger at the time of breast cancer diagnosis (p<0.001). Patients with triple-negative disease had relatively higher rate (17.5%) and mostly in *BRCA1/2* genes (71.4%) (26).

## 3 Patients at risk

Several international guidelines, including the American Society of Clinical Oncology (ASCO) (27), the NCCN (28), the American Society for Radiation Oncology (ASTRO) (29), and the European Society for Medical Oncology (ESMO) (30), attempted to select patients at higher risk for carrying PV/LPVs. Most of these guidelines were based on consensus, and not a result of randomized clinical trials. The NCCN guidelines are updated frequently and often such updates might not be closely followed by practicing community oncologists. The most recent criteria were expanded to include older patients (50 instead of 40 years), and all patients with triple negative disease regardless of their age (**Table 1**). However, the recent introduction of poly ADP ribose polymerase (PARP) inhibitors to treat patients with *BRCA1/2* variants resulted in more expansion of the testing guidelines to include all patients who may potentially benefit from certain anti-cancer therapy used in the setting of *BRCA1/2* variants. A randomized phase-3 trial (OlympiAD) showed that olaparib, a PARP inhibitor, when compared to palliative chemotherapy, in human epidermal growth factor receptor 2 (HER2)-negative metastatic breast cancer patients, with pathogenic germline *BRCA1/2* variants, was associated with better progression-free survival (PFS) (31). Similar results were reported using talazoparib, another PARP inhibitor (32). More recently, PARP inhibitors were also tried in the setting of high-risk early-stage breast cancer with germline pathogenic *BRCA1/2* variants (Olympia trial). When compared to placebo, adjuvant olaparib for one year was associated with significant improvement in distant (dDFS) and invasive (iDFS), disease-free survivals, and possibly overall survival (OS), too (33).

**Table 1:** Recommendations for germline genetic testing*.

Age^^^	Gender^^^	Ancestry^^^	Treatment^^^ Indication	Pathology^^^	Family History^^^
All patients ≤ 50 years	All male patients	All patients with Ashkenazi Jewish Ancestry	Systemic treatment decisions using PARP inhibitors for MBC	Triple-negative breast cancer	Breast cancer at age ≤50 years
					Male breast cancer
				Multiple primary breast cancers (synchronous or metachronous)	Ovarian cancer
			Adjuvant treatment decisions with olaparib for high-risk, HER2-negative EBC		Pancreatic cancer
				Lobular breast cancer with personal or family history of diffuse gastric cancer	Prostate cancer with metastatic, or high- or very-high-risk group
					≥3 Total diagnoses of breast cancer in patient and/or close blood relatives
					≥2 Close blood relatives with either breast or prostate cancer (any grade)

*As per the National Comprehensive Cancer Network (NCCN) guidelines.

^ Regardless of any other risk factor.

PARP, Poly ADP ribose polymerase; MBC, Metastatic breast cancer; HER2, Human epidermal growth factor receptor-2; EBC, Early breast cancer.

Given this expansion in the indications for genetic testing, it’s estimated that almost two-thirds of breast cancer patients will have at least one indication for genetic testing. However, many studies had shown that the current testing guidelines are restrictive and only a fraction of eligible patients are tested (34, 35). Additionally, several other studies had shown that the prevalence of PV/LPVs in the other non-tested patients are high enough to justify testing all patients in a testing approach known as “universal testing” (36). This approach was adopted by the American Society of Breast Surgeons, which called for testing all breast cancer patients regardless of their age, personal or family history of cancer.^37^

## 4 Surgery for the diseased breast

Options for the diseased breast varies and can range from BCS (followed by radiation therapy) to many forms of mastectomies. Each option has its own advantages and obviously some potential setbacks (37).

### 4.1 BCS versus mastectomy

Tumor’s characteristics, including size and site, and patient’s characteristics, like breast size, may determine the extent of surgery; mastectomy versus BCS, regardless of the existence of *BRCA1/2* variants. Patients with newly diagnosed early-stage breast cancer who carry a PV/LPV in *BRCA1* or *BRCA2* are often advised to undergo mastectomy, which can be skin-sparing or nipple-sparing. BCS was never compared, in a randomized study, to mastectomy in this setting. Much of our knowledge, however, is based on small retrospective studies and pooled analysis of such studies.

In one systematic review that included 3,807 patients in 23 observational studies, differences in outcomes between mastectomy and BCS among breast cancer patients with *BRCA1/2* variants were analyzed. Patients were young with a median age at breast cancer diagnosis of 41 years; 2,200 (57.7%) had *BRCA1* variants while 1,212 (31.8%) had *BRCA2*. BCS was performed on 2,157 (56.7%) while 1,408 (41.5%) patients had mastectomy. Risk of loco-regional relapse (LRR) was significantly higher in the BCS group (HR: 4.54, 95% CI: 2.77-7.42, p<0.001). However, disease-specific recurrence (HR: 1.58, 95% CI: 0.79-3.15, p=0.200), disease recurrence (HR: 1.16, 95% CI: 0.78-1.72, p=0.470), contralateral breast cancer (HR: 1.51, 95% CI: 0.44-5.11, p=0.510), and death (HR: 1.10, 95% CI: 0.72-1.69, p= 0.660) were not higher in the group who underwent BCS (38).

In another systematic review of 18 studies that compared BCS and mastectomy, OS at 5, 10, and 15 years were comparable (83%, 86.0%, and 83.2%) with mastectomy, and with BCS (88.7%, 89.0% and 83.6%), respectively. However, the ipsilateral breast cancer recurrence rates at 5, 10, and 15 years were significantly lower with mastectomy (3.4%, 4.9%, and 6.4%, respectively) than with BCS group (8.2%, 15.5%, and 23%, respectively). Researchers concluded that BCS can be offered for select patients with *BRCA1/2* mutation after proper counseling and with intensive follow-up (39).

Patient’s satisfaction for cosmetic results should always be balanced against oncological safety. The need for adjuvant radiation therapy following BCS and the possible increase in the risk of complications that may lead to a possible subsequent mastectomy with immediate breast reconstruction should always be addressed with patients when considering BCS versus mastectomy.

### 4.2 BCS in *BRCA1/2* vs sporadic breast cancer

Several other studies had attempted to answer the question of the oncological safety of BCS by comparing the outcomes of patients with *BRCA1/2* mutation to a control group of patients with sporadic breast cancer. In one retrospective study that reviewed the clinical and pathological records of 501 patients who underwent BCS in China between 2005 and 2018, 63 patients had *BRCA1* or *BRCA2* variants. After a median follow-up of 61 months for carriers and 70 months for noncarriers, the DFS (p=0.424) and the OS (p=0.173) were not significantly different. Interestingly, there was no difference between the two groups in ipsilateral breast tumor recurrence (p=0.348). However, CBC was significantly worse in carriers; 9.5% versus 0.68%, p<0.001 (40). No significant difference in ipsilateral-breast tumor recurrence (IBTR) was also reported in another Chinese study (41).

In another meta-analysis that included 13 studies with 701 BRCA-mutation carriers and 4,788 controls, IBTR was significantly higher in BRCA-mutation carriers (RR: 1.589; 95% CI 1.247-2.024; p<0.001). As expected, risk of recurrence increased as the follow up increases; (RR: 1.601; 95% CI 1.201-2.132) with 10 or more years of follow up and (RR: 1.505; 95% CI 1.184-1.913) with median follow up of 7 or more years. However, overall survival in three included cohort studies found no evidence to suggest a deterioration in OS in patients with BCS (38). Multiple other studies had confirmed the high rate of IBTR in *BRCA1/2* carriers treated with BCS compared to matched controls with sporadic breast cancer (42).

## 5 Risk-reducing mastectomy

Compared with non-carriers, patients with *BRCA1/2* mutation have a higher risk for contralateral breast cancer with *BRCA1*-mutation is associated with higher risk compared to those with *BRCA2*. Several studies had compared outcomes of women who underwent risk-reducing mastectomies with those who opted to continue on surveillance (43). Surgical decision-making process is quite complex and should take into consideration several risk-modifying factors including age at first breast cancer diagnosis, the use of adjuvant endocrine therapy and planned, or already performed oophorectomy. Younger patients who have not received adjuvant endocrine therapy or undergone oophorectomy, might be at higher risk for ipsilateral breast cancer recurrence (IBCR) and CBC, and thus might benefit from a more aggressive surgical approach. Women with strong family history, like those with family member diagnosed or died, with breast cancer at younger age, tend to choose mastectomy, while younger patients aged 30 or less are more likely to choose surveillance. Anxiety and fear of getting a second breast cancer are significantly lower following RRM, which impacts positively on the quality of life of such patients (44). Several surgical options are available to manage the contralateral breast but mostly nipple-sparing, skin-sparing mastectomy, which is usually associated with excellent cosmetic and oncological results.

### 5.1 Skin-sparing and nipple-sparing mastectomies: how effective and how safe?

In skin-Sparing mastectomy (SSM), a radial, axillary or an inframammary incision is utilized, much of the breast skin is spared but carefully dissected off breast tissue with removal of the entire breast glands to create a pocket that facilitates immediate breast reconstruction with implant or autologous graft. Nipple-sparing mastectomy (NSM) is similar to SSM, but the nipple-areola complex (NAC) is preserved, as well (45, 46). Both techniques are increasingly utilized in clinical practice and are associated with superior cosmetic outcomes and better patients’ satisfaction compared to mastectomy (47–50). In addition to the usual complication encountered with other types of breast reconstructions, NAC necrosis is the main complication of NSM and tends to be higher among smokers, obese and those with large breasts, and following radiotherapy (51, 52).

However, one of the main concerns associated with both SSM and NSM is the risk of local breast cancer recurrence at the NAC secondary to occult nipple involvement or a second new primary cancer in the retained breast tissue (53–57). Such risk is obviously higher among patients who carry a pathogenic germline breast cancer predisposing genes. Breast cancer recurrence at the NAC, often referred to as “oncologic safety” can be a concern. Several studies, mostly retrospective ones, attempted to answer the question in two groups; the affected patients who underwent contralateral prophylactic surgery, and among unaffected carriers.

The oncologic safety of SSM and NSM was initially studied in the setting of sporadic breast cancer. In a 2010 meta-analysis of 9 studies that enrolled 3,739 patients, rates of local recurrence in SSM did not differ significantly from those who underwent non-SSM (53). Another meta-analysis of 20 studies involving 5,594 women with early-stage breast cancer did not detect any differences in local recurrence, DFS or OS between those receiving SSM compared to those receiving conventional mastectomy without reconstruction (54). Another large systematic review of 17 retrospective studies included 7,107 patients; majority (85.4%) of them had the procedure for invasive carcinoma. Following a median follow up of 48 months (range 25-94), the mean rates of local recurrence was 5.4% (0.9-11.9), and recurrence involving the NAC was 1.3% (0-4.9) (55). Another large retrospective study from Korea that involved 944 patients, reached similar conclusions. Multicentricity or multifocality, negative hormone receptor, or HER2-positive subtype, high histologic grade, and extensive intraductal component, were independently associated with cancer recurrence at the NAC after NSM (56).

Several other studies addressed issues related to oncologic safety among patients harboring a pathogenic cancer-predisposing gene. In one study, researchers examined tissues from 62 NACs from 33 women (25 *BRCA1*, 8 *BRCA2*) who underwent mastectomy between 1987 and 2009 at Mayo Clinic. Atypical hyperplasia, carcinoma in situ, or invasive carcinoma were not found in any of the 33 prophylactic mastectomy specimens performed. However, 2 (7%) of the 29 breasts with cancer, and available tissue, had malignant findings, and 1 (3%) had atypia in the NAC (57).

More recently, Rocco et al. reviewed 9 studies reported on the incidence of primary breast cancer following NSM in *BRCA1/2* unaffected carriers who undergo prophylactic bilateral mastectomy. From an oncological point of view, NSM appears to be a safe option for BRCA mutation carriers, with low reported rates of new breast cancers. Additionally, the procedure was associated with low rates of postoperative complications, and high levels of satisfaction and postoperative quality of life (58). In another study, researchers reviewed 114 NSM performed from 2008 to 2019 on patients with breast cancer in 105 *BRCA1/2* carriers (56 *BRCA1*, 47 *BRCA2*, and two women with both mutations). Five (4.4%) patients had positive nipple margins on final pathology and all underwent nipple excision. Systemic therapy was offered to 76% patients; 65 (62%) with chemotherapy and 48 (46%) received endocrine therapy. Patients were followed up for a median of 70 months (range 15-150), no patient had a recurrence in the retained NAC or at the site of a nipple excised for a positive margin. The rate of locoregional recurrence outside the nipple and distant recurrence were also low at 2.6% and 3.8%, respectively (59).

In another study from 9 major institutions in the US, researchers retrospectively reviewed their experience on 548 prophylactic NSM performed in a cohort of 346 patients with *BRCA1* or *BRCA2* variants. Unilateral risk-reducing NSM secondary to a concurrent, or prior cancer in the contralateral breast, were performed on 144 (41.6%) patients, while bilateral prophylactic NSM were performed on 202 (58.4%) patients. With median and mean follow-up of 34 and 56 months, respectively, no ipsilateral breast cancers were reported after prophylactic NSM. Similarly, breast cancer did not occur in any patients undergoing bilateral risk-reducing NSM (60).

## 6 Moderate penetrance genes

**Figure 1:**
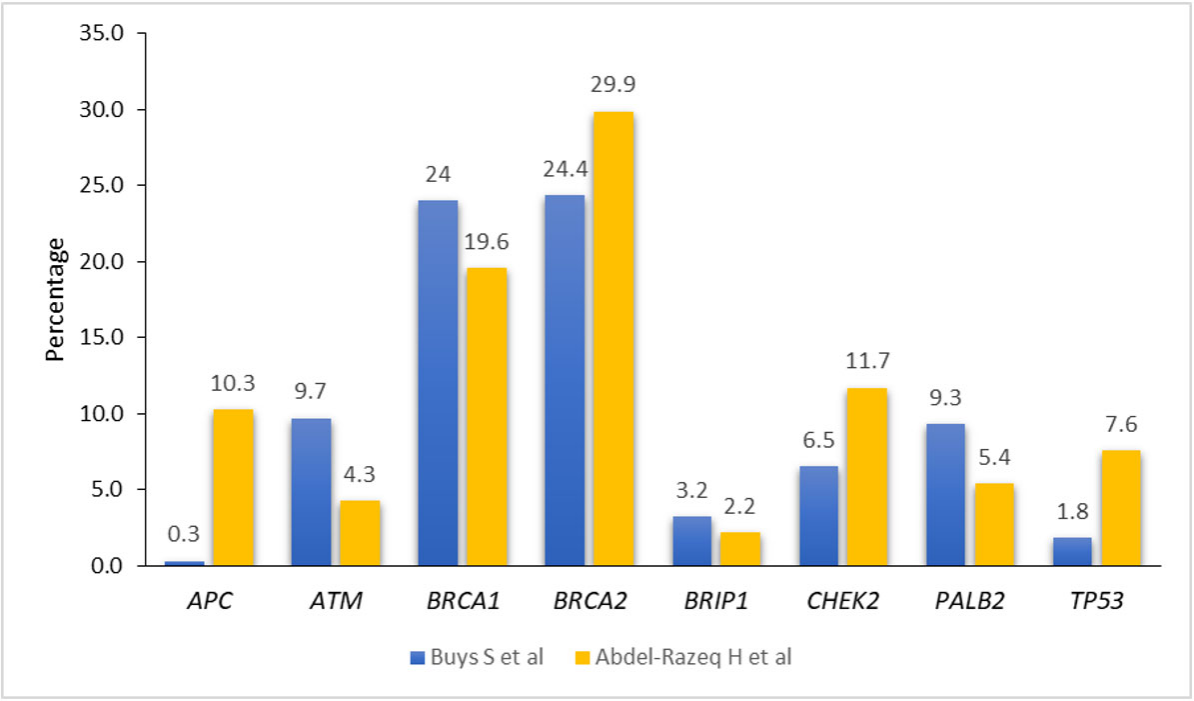
Prevalence of pathogenic/likely pathogenic variants among breast cancer patients in different ethnic groups.

The recent advances in NGS technologies resulted in an increase use of multigene panel testing and enabled sequencing of *BRCA1/2* concomitantly with many additional genes. Recent studies suggest that other cancer predisposing genes, including *PALB2, ATM, CHEK2, TP53, RAD51C*, *RAD51D*, and many others, confer variable risks of breast and other cancers (61–63). Rates of such variants are very variable, depending on population studied and testing method utilized. **Figure 1** illustrates an example of such variation in a study that used a 25-multi gene panel, and enrolled over 35,000 patients; half of them were non-Western with different ethnic background (24), and a recently published study from our group that enrolled over 1,000 Arab breast cancer patients utilizing a multi-gene panel, too (26). Appropriate counselling and data-driven risk management with appropriate plans for risk-reducing intervention or surveillance for patients with breast cancer and unaffected individuals, are highly needed (64–67).

### 6.1 PALB2

Pathogenic/likely pathogenic *PALB2* variants is associated with high risk for breast cancer, with studies showing a life-time risk of 40-60% (68). One multi-national study that analyzed data from 524 families with *PALB2* PVs in 21 countries concluded that the estimated relative risk (RR) of breast cancer was 7.18 (95% CI, 5.82- 8.85; p=6.5×10^-76^) (69). A large family-based study reached similar conclusions (70). Additionally, patients harboring PVs of *PALB2* are at higher risk for ovarian cancer and Fanconi anemia which is inherited in an autosomal recessive manner (71). The NCCN guidelines recommend annual mammogram beginning at age 30 years with consideration for breast MRI. Risk-reducing surgery should also be discussed with the patient.

### 6.2 CHEK2

The rate of *CHEK2* germline mutation is higher in certain ethnic groups like the Northern European countries. Certain variants in the *CHEK2* gene (I157T and c.1100delC) are associated with higher risk for breast cancer (72). The cumulative lifetime risk ranges from 28% to 37% (73). While no data available on the benefit of RRM, annual mammogram and breast MRI once a year starting at 40 years of age, are highly recommended. Carriers of *CHEK2* pathogenic variants are at higher risk for colon, prostate, bladder, kidney and thyroid cancers, more so with c1100delC variant (74).

### 6.3 TP53

The *P53* is a tumor suppressor gene that prevents the development of cancer. Patients with germline mutation, Li-Fraumeni syndrome, are at risk for early-onset breast cancer, sarcomas, and other cancers in children and young adults (75, 76). Following cellular stress, like radiation therapy (RT)-associated cell injury, *P53* provides the cell with ability to repair DNA damage through multiple downstream repair pathways. In a small series of 8 patients with breast cancer and germline *TP53* pathogenic variant, 6 of them were treated with radiation therapy following surgery, ipsilateral breast recurrences were reported in three and contralateral breast cancers in three more. RT-induced cancers were reported in two, in addition to three new primary cancers. On the other hand, only one contralateral breast cancer occurred among patients who had not received radiation therapy (77). Several other case reports of RT-associated malignancies supported the recommendation against RT in patients with *TP53* (78–82). As such, mastectomy should be recommended to possibly avoid radiation therapy following BCS.

### 6.4 ATM

Heterozygous pathogenic variant in *ATM* is associated with a 13-33% cumulative lifetime risk for breast cancer (83, 84). Risk-reducing mastectomy is not recommended for carriers; however, it might be considered based on personal and family history. No apparent risk of post-surgery radiation therapy on patients with pathogenic variant. Mammogram with consideration of breast MRI is recommended yearly starting at age 40 years.

## 7 Conclusions

Germline genetic testing is currently offered for majority of patients with breast cancer, as it informs both preventive and treatment decisions. Available data support the oncologic safety of more conservative surgical approaches in breast cancer patients even with the highest penetrant germline variants like *BRCA1* and *BRCA2*. Unaffected carriers may also be offered active surveillance should they choose so. However, evidence to guide clinical decisions on less frequent, mild to moderate risk variants, is lacking.

## Author contributions

HA-R: Conceptualization, Data curation, Methodology, Project administration, Supervision, Writing – original draft, Writing – review & editing.
